# Perceptions and experiences of access to public healthcare by people with disabilities and older people in Uganda

**DOI:** 10.1186/s12939-014-0076-4

**Published:** 2014-10-08

**Authors:** Moses Mulumba, Juliana Nantaba, Claire E Brolan, Ana Lorena Ruano, Katie Brooker, Rachel Hammonds

**Affiliations:** Center for Health, Human Rights and Development, Kampala, Uganda; School of Population Health, The University of Queensland, Brisbane, Australia; Department of Global Public Health and Primary Care, Center for International Health, University of Bergen, Centro de Estudios para la Equidad y Gobernanza en los Sistemas de Salud, Bergen, Guatemala Guatemala; Queensland Centre for Intellectual and Developmental Disability, The University of Queensland, Brisbane, Australia; Institute of Tropical Medicine, Antwerp, Belgium

**Keywords:** Africa, Uganda, Persons living with disabilities, Older persons, Millennium development goals, Sustainable development goals, Go4Health, Human rights

## Abstract

**Introduction:**

In the year 2000, a set of eight Millennium Development Goals (MDGs) were presented as a way to channel global efforts into the reduction of poverty and the promotion of social development. A global discussion regarding how to renew these goals is underway and it is in this context that the Goals and Governance for Global Health (Go4Health) research consortium conducted consultations with marginalized communities in Asia, Latin America, the Pacific and Africa as a way to include their voices in world’s new development agenda. The goal of this paper is to present the findings of the consultations carried out in Uganda with two groups within low-resource settings: older people and people living with disabilities.

**Methods:**

This qualitative study used focus group discussions and key informant interviews with older people in Uganda’s Kamwenge district, and with persons with disabilities from the Gulu region. Thematic analysis was performed and emerging categories and themes identified and presented in the findings.

**Findings:**

Our findings show that a sense of community marginalization is present within both older persons and persons living with disabilities. These groups report experiencing political sidelining, discrimination and inequitable access to health services. This is seen as the key reason for their poor health. Clinical services were found to be of low quality with little or no access to facilities, trained personnel, and drugs and there are no rehabilitative or mental health services available.

**Conclusion:**

Uganda must fulfil its international obligations and take progressive measures to meet the right to health for all its peoples, but especially allocate its limited resources to proactively support its most marginalized citizens. The growing impetus within post-2015 development negotiations to redress in-country health and other inequalities through a comprehensive systems approach is of importance in the Ugandan development context. This approach reflects the participant’s perspectives, which also calls for a more equitable approach to health and development as opposed to a narrow, vertical focus on specific population groups, as was the case with the MDGs.

## Introduction

Today, people with disabilities represent 15% of the world’s population, or around one billion people, 80% of which live in low and middle-income countries [1]. Conflict and war, rapid population growth, increasing life expectancies, the increasing non-communicable disease burden, and the ageing process have all contributed to an increased prevalence of disabilities, while the lack of responsive policies that allow people to realize their rights in regards to education, employment and healthcare provision means that disability places individuals and families at greater risk of experiencing poverty [1,2]. In addition, as the number of older people (adults over 60 years) will increase to an expected two billion people by 2050, many countries now face the challenge of caring for an ageing population [3]. Neither people with disabilities or older people are homogenous groups. Individuals in these two population groups experience differing degrees of vulnerability; many experience complex economic and social challenges related to income insecurity, poverty, mobility, stigma and environmental degradation. These factors are compounded by the rise in non-communicable diseases, which can affect people with disabilities and older people disproportionately, and the risk of infectious diseases like HIV/AIDS [4-6].

Uganda has a population of 36 million people, a number that is projected to increase five-fold by the year 2100, and is one of eight countries expected to account for over half the world’s projected population before the end of the century [7]. Named one of the least developed countries in the world in 2013, and located in one of the less developed global regions, it has seen the life expectancy of its population grow from 43 years in 1990 to 56 in 2011 [8]. This translates into a growing number of older people that will require access to economically productive activities as well as to health and other social services. Alternatively, approximately 19% of the Ugandan population over five years of age lives with at least one disability. The prevalence of disability in Uganda increases with age from 12% for children 5–9 years old to 67% for those over age sixty [8]. Uganda is a State party to numerous international human rights treaties, including the International Covenant on Economic, Social and Cultural Rights (CESR), enshrining numerous rights including the right to health and the recent United Nations Convention on the Rights of Persons with Disabilities (CRPD) [9]. In acceding to these treaties Uganda pledged to realize the human rights of vulnerable and marginalized groups including older people and people with disabilities.

In the year 2000, a set of eight Millennium Development Goals (MDGs) were presented as a way to channel global efforts into the reduction of poverty, the improvement of access to education, of gender equity and of the reduction of certain disease burdens, all by the year 2015 [10-12]. As the end-date for the MDGs nears, and as country and global reports show a number of the goals will not be met, the future of global health and development policies needs to be considered [13]. The Goals and Governance for Health (Go4Health) research consortium has a mandate from the European Commission to provide policy recommendations for the new post-2015 health and development goals. In order to do this, Go4Health proposes a global governance structure that includes the voices of communities that have not previously been a part of shaping health policy at this level [14]. A multi-sited, qualitative research project was implemented in countries in Asia, Africa, Latin America and the Pacific region with the goal to give voice to people who are not typically consulted or heard. This multi-region study engaged the voices of marginalized and excluded communities on the post-2015 development agenda. The purpose of the consultations was to identify health needs, barriers and issues from communities and build on them in order to provide recommendations into the larger, post-2015 global discussion regarding the content of new health and development goals. In Africa, this project was led by the Center for Health, Human Rights and Development (CEHURD).

In many regions of the world there are populations that are either excluded from policy processes or are unable to actively engage in them. When this is combined with historical, social, political and economic determinants, it can lead to whole communities being disenfranchised from their governments [15]. As a State party to numerous human rights treaties the Ugandan state is obligated under international human rights law to include marginalized populations in the national policy process and prioritize their concerns in post-2015 sustainable development goal discussions. This article reports on a sub-study within Go4Health that was carried out in Uganda in 2013. The fieldwork took place with people with disabilities living in the post-conflict district of Gulu, in the north of the country, and with older people living in the isolated western district of Kamwenge. The aim of this study was to gain a deeper understanding of the perceptions and experiences of both groups around accessing public healthcare and inter-related social services with the goal of feeding into Go4Health’s broader policy recommendations on the new post-2015 health development goals.

## Methods

### Setting

#### People with disabilities – Gulu district

To elicit the perceptions and experiences of people with disabilities, a collaboration between CEHURD and the non-government organization, Gulu Disabled Persons Union, was formed in the Gulu district of Uganda. This district is located five hours north of Kampala, the capital of Uganda. The Uganda census in 2002 reported the population of Gulu district to include 475,260 people. In regards to health infrastructure, Gulu has 45 government health facilities, including 26 level two units, 14 level-three health units, 3 level-four health centers, one general hospital and one regional referral hospital. This district was selected to participate in this study because it is recovering from a long civil war that resulted into violence with emerging disabilities of various forms.

Gulu has experienced the longest civil conflict in the history of Uganda, a 20 yearlong war between the Lord’s Resistance Army (LRA) and the Ugandan government. Since the peace signing between the two factions in 2006, the district has been considered a post-conflict environment [16]. The conflict has had a devastating effect on the lifestyle, health and well-being of the people of Gulu; communities were subjected to rape and gender violence, torture, injuries due to landmines and weapons, mass abductions, geographical displacement, and murder [17,18]. Currently, the region is rebuilding itself, with special efforts on improving security, infrastructure and the lives of the people in the many affected communities [19,20]. However, Gulu’s poor economy has hampered efforts [21]. As a result, the majority of the population lives in poverty and 46% of the total population experiences food insecurity. The people of the district are engaged mostly in small-scale business, which are mainly for day-to-day survival [21,22]. Although there have been no studies that clearly state how many people experience disabilities in Gulu as a result of the conflict (or otherwise), the prevalence is thought to have risen significantly due to the use of landmines and because of the magnitude and scope of the fighting [23,24].

#### Older people – Kamwenge district

To elicit the perceptions and experiences of older people, CEHURD worked in collaboration with volunteers for community development from the Kanara Village health team, as well as with representatives from the local government. Kamwenge has no special organization for older people. The district is located more than ten hours west from Kamapala, and in 2009 it had an estimated population of 309,700 people with an estimated rate of population growth of 2.9% [8]. The district does not have a hospital, but it has two sub-health districts with level 4 health units, and 33 health units that allow for coverage for 44% of the 75 parishes. In regards to human resources for health, out of 343 posts that were approved for the district, only 195 are filled. This leaves 148 posts vacant. This has led to Kamwenge having two health sub-districts that do no have any medical officers.

This district was selected to participate in the study because of its remote location in regards to the capital city and the large population of older persons, who represent 8.5% of the population [8]. The geographical isolation that characterizes this district and the marginalization of its population means that local communities experience high levels of poverty and other socioeconomic deprivations. The majority of the population relies on subsistence farming and cattle-raising for their livelihood. Crops grown include bananas, maize, beans, cassava, groundnuts and potatoes.

### Data collection and analysis

A qualitative study was conducted using a participatory action research approach to seek and understand the public health service in Uganda for people with disabilities and older people. A participatory approach was used and defined as a reflective process which researchers and participants undergo together to understand the research area [25,26]. Researchers worked with members of the communities in Gulu and Kamwenge. In engaging with both of the selected communities three sequential steps, detailed below, were used to inform subsequent actions. Due to project constraints, our approach was focused primarily on working with community leaders [26].

The first step involved eliciting reflections from local leaders, community members and representatives from political, religious and other institutions in the districts. The aim of the reflections was to highlight areas of importance for the community and to inform areas of discussion in the later actions of the research. The reflections were focused on health-related priorities and needs, as well as healthcare challenges and inequities. Through these discussions, rapport was established and trust built in a way that allowed us to work more deeply with local communities [25,26]. Afterwards, a discussion guide and an interview guide were developed, guided by the reflections that resulted from our participative approach and from the Go4Health domains of inquiry: community understanding of health, social determinants of health, roles and responsibilities of relevant stakeholders and community participation in decision-making [14]. This guide was used in the second and third steps.

The second step involved focus group discussions with the selected communities in each district. One focus group discussion (FDG) was conducted in each district. The topics for the FDGs were based on the discussion guide produced during the first action. Areas of discussion included the health-related priorities and needs as highlighted by community leaders, community understandings of health and disability, the social determinants of health, the roles of community leaders in healthcare and the level of community participation in decision-making for people with disabilities and older people.

Finally, the third step involved conducting key informant interviews with local leaders in each district regarding the specific health needs and the challenges experienced by the people with disabilities and older people. The interview guide used in the key informant interviews was developed in the first action. Areas of discussion included the health-related priorities and needs as highlighted by community leaders in the first action, the roles of community leaders in healthcare, community understandings of health and disability and the level of community participation in decision-making.

The key informant interviews and focus group discussions were conducted in the local language by trained interviewers. Afterwards, the transcripts were translated into English and transcribed verbatim. The transcripts were analyzed using thematic analysis, a method that allowed for the identification of recurrent patterns that can be grouped into categories and themes and represented a recursive process [27]. The transcripts were analyzed by ALR and CEB. The first step was to carefully read the transcriptions in order to become familiar with the data. The data was summarized and a list of the initial codes and themes was developed. The international research team discussed the themes during meetings and over Skype and electronic mail. The entire dataset was then systematically coded. The initial set of codes was revised and clusters and later categories that were grounded in the text were developed. These categories were grouped into themes. Specifics of each theme were refined in an ongoing analysis of the data and definitions of each theme were generated [27,28]. Finally, quotes were selected for their capacity to exemplify the main message of each of the five themes.

### Ethical considerations

Prior to commencing the study, ethical clearance was sought from Uganda’s National Council of Science and Technology where CEHURD submitted the research proposal and protocol. The National Council of Science and Technology issued a letter to the Residence District Commissioner of the districts informing them of the research project. The research team introduced themselves to the local government leadership as part of the ethical clearance process. Permission from the local government leadership was granted for the research in the communities in both districts. The research team worked closely with community based organizations that worked intimately with both population groups in the two districts.

The nature of disability and literacy level were taken into consideration for all participants. Efforts were made to ensure the participants fully understood and provided their consent to the consultation process. Research was collected in the local language and was used together with English where necessary. Interviews with people with disabilities were conducted in accessible venues in Gulu. These venues were where meetings with this population group generally took place in the community. The participants were assured of their right to withdraw from the study at any time without giving a reason, and without any consequences. Anonymity and confidentiality was ensured in order to protect participants’ identity.

## Findings

### Theme 1: Marginalized and Stigmatized: ‘Most of them see us as monkeys’

Marginalization and stigma were common feelings reported by the study participants from both districts. These stemmed, in part, from the feeling that their communities rejected them because of their disability or age. The rejection would translate into negative health outcomes with the stigma of being ‘less than their neighbors’ impacting not only on the health of the people, but also the way they lead their daily lives. Participants reported elevated levels of stress and anxiety. Participants also reported feelings of not belonging to their community or families. The stigma that came with disability had a strong impact on the individual, regardless of how the disability came to be, as one participant recounted:

*‘When somebody gives birth to a disabled child, that child is not treated the same as other children and they are segregated and usually marginalized. This does not stop there but persists into later states of life. This greatly affects the health of disabled people’.*

In this study, participants viewed their disability as a barrier to good health. The disability participants experienced made the enjoyment of everyday life and activities difficult and hindered their capacity as individuals to become truly active in community life. The participants reported the discrimination they experienced was very much entrenched in the local community and culture. The discrimination both groups of participants faced was perpetuated by the attitudes of community members, who they perceived treated people with disabilities and older people as ‘*worthless’* and as a *‘waste of time’*. This led to feelings of despair, and to the perception that, as one community member shared:

*‘This* [the way people with disabilities are treated in their communities] *is the result of ignorance from the public on the fact that we are also able to perform and work as others. Most of them see us as monkeys on trees’.*

### Theme 2: Political Marginalization: ‘It’s like we never existed’

Participants with disabilities and older participants felt the discrimination they faced went beyond what they encountered in their communities, and expressed feelings of marginalization from broader political life. Participants with disabilities expressed that the government, both local and national, had never taken actions to incorporate them into any sort of participatory process. A participant from the Gulu district stated:

*‘Persons with disabilities participating in planning, implementation and monitoring… is low. Though we try to participate in different activities, it is still low. This is because of lack of information to persons with disabilities on what is taking place and also mobility to reach places where such activities are taking place is low’.*

Alternatively, older participants reported feeling the same exclusion from government-related activities as participants with disabilities, and attributed the exclusion to their age. Many older participants from Kamwenge felt abandoned by the government or the state, and this stemmed from seeing many political promises that never materialized. At the community level, participants reported that community leaders only approached older people when it was for their own personal gain:

*‘The community leaders don’t care about us. They never invite us to their meetings or even inquire from us… it’s like we never existed… the leaders only come during election time… the community leaders go and tell us to form committees for old people, persons with disabilities, youths, we do so… they even bothered us to make a constitution, we got a certificate and then they asked us for money –asked us to make circles- we paid! But we have never heard from them until now. We have never got money or heard from them since’.*

When asked why older people were not being prioritized by the government, participants offered two reasons. First, there are too few older people in the country, and this means their votes count for only a small percentage and therefore mean very little to politicians. Second, the feeling was the government cares for children because they are many and by catering to them, they also gain the support of the children’s parents. The neglect felt from the central government fostered feelings of anger, let down and political isolation. As one participant stated:

*‘They chased us* [old people] *out of the district councils, it’s like we are not people, and no one makes us a priority in the budget’.*

### Theme 3: Inequity in health: ‘Different levels of healthy’

Both groups of participants reported being aware of the differing levels of health they experience when compared to the general population. Members from both communities identified poor nutrition and lack of food as a problem. However, inequity in health status and access to nutritious food was particularly important for participants from Gulu. Participants with disabilities reported that some people with disabilities, particularly those unable to work in the fields or materially contribute to the family income, were usually given less food or food with lower nutritional value. When it comes to health, this translates into a fragmentation of what healthy means. As one participant from Gulu stated:

*‘I think there are different levels of healthy, and we as disabled persons, we have the lowest level of health’.*

The participants from Kamwenge shared this feeling, and one older person stated:

*‘As an older person I have no good health, I have no peace at all and old age is bad health’.*

The two communities identified different health-related problems and causes for their ill health. For participants with disabilities, HIV/AIDS played a key role in regards to their health status. Being HIV positive meant they needed constant medication and were always at risk of developing infections or tuberculosis. Receiving treatment for this was complicated, as it meant travelling long distances. In addition to mobility, being mistreated by health workers was another deterrent from accessing both preventive and curative care:

*‘For instance, if a blind person goes for a HIV/AIDS test, instead of helping that person, the health workers instead ridicule that person and ask them ‘even you with your blindness, how could you get someone to give you HIV/AIDS?’ this discourages us from testing and so most of us don’t know our HIV/AIDS status’.*

Another reason for differing health given by participants related to the mix of vulnerability and poverty of local people with disabilities. Participants considered some local people with disabilities tended to enter into sexual relationships as a means to survive, something that placed them at higher risk for developing HIV/AIDS.

In contrast, older participants did not think of exposure to HIV/AIDS as problematic for them in the same way that malaria and syphilis were. For this group, most health problems they identified resulted from poor hygiene, sanitation, and a lack of safe and good quality water. For older participants, education was the key to getting local people to accept and implement preventive strategies.

Finally, infrastructure also played a major role when it came to the feelings of what being healthy is for people with disabilities. A lack of access to assistive devices, a lack of an enabling environment with ramps and clear signaling or safe roads contributed to the acquisition and spread of disease in Gulu. One participant shared:

*‘Though some persons with disabilities have wheelchairs or crutches, others don’t and walk on their hands and knees, so there is poor sanitation and the sanitation facilities have not been customized for people with disabilities. Persons with disabilities end up with diseases as a result of poor hygiene and sanitation’.*

#### Promoting socioeconomic opportunity and good health

In reflecting on their lives and health-related experiences, older participants from Kamwenge emphasized the key role that education could play in moving out of poverty and into a place where there were more economic opportunities. This would lead to better health. Participants perceived that there was a clear causal relationship between being poor and lacking an education, and this directly impacting the health of the people. As a participant stated:

*‘God gave us different incomes, different lands, those with good land can cultivate, take their children to schools and be rich. Being rich and poor causes the difference in health statuses’.*

Older participants saw the cycle of poverty, lack of education and poor health repeating itself in the next generation of local people: ‘*If parents don’t have money, they will not be able to take the children to school and the work in the fields will make children sick’.*

### Theme 4: Lack of access to appropriate medicine and health services

Participants with disabilities reported experiencing discrimination that impacted on their ability to access health services and medicines, an infringement of the right to health, and other human rights breaches by healthcare workers. In addition, their family members also received ill treatment and, at least in one case, sexual abuse. One participant elaborated:

*‘In other cases where a man with a disability goes to the hospital with his wife to seek medical attention… the health workers in some cases refuse to work on them or avail them with medicines unless their women have sexual intercourse with them’.*

The older participants did not raise reports of physical and/or sexual abuse, but they did identify the devaluing attitudes of health workers towards them. This included a sense of neglect some received from healthcare staff because of their age, which acted as a barrier to accessing appropriate healthcare. It was this group of participants who stated that the main barriers they faced when visiting hospitals were not being informed of their condition or not being provided with a diagnosis. The lack of adequate care and medication led many to visit local herbalists. As a participant stated:

*‘We get prescriptions but cannot afford to buy the medicines. You walk to the health center, they write you a paper. Now, where to we get the money to buy the medicines?’*

While older participants focused on the relationship between education and health, participants with disabilities discussed issues of breaches of sexual and reproductive rights. For example, it was reported that health services did not have adjustable hospital beds in delivery wards for women giving birth, and communication barriers between physicians and their patients with disabilities results in life-threatening situations. A woman from Gulu shared:

*‘There are some of us who are deaf and for the pregnant women, when they go to the health centers to give birth, they have problems because there is no way for the medical people to communicate with them, which results in the death of their children as they don’t understand the instructions of the midwives and medical people’.*

Another woman from Gulu highlighted:

*‘When I was pregnant, I went to the health center for antenatal care but the medical personnel could not take my health history, because I am deaf and dumb, and though I know sign language, there was nobody who could understand what I was saying and I could not understand the health education session given to the other pregnant women. This was very frustrating as the doctor was just estimating everything and writing his own things. This put my life and that of my baby in danger’.*

### Theme 5: The need to expand quality rehabilitative and mental health services

The participants with disabilities resided in a post-conflict environment and they expressed a need for targeted trauma counseling services. This care should be affordable and sustainable, and the services should provide assistive devices and rehabilitative services such as support for amputees and others experiencing disability as a result of the conflict. One participant stated:

*‘I use a wheelchair but at times it breaks down. The chair was a donation but when it breaks down, I have no means to repair it because it is very expensive to carry out repairs and what I earn cannot cover this’.*

The negative impact the conflict had on the psychological and emotional health and wellbeing of people with disabilities resulted in many participants experiencing psychological issues. Abuse of alcohol was reported as common and was seen as a coping mechanism. Participants considered alcohol abuse as a viable option and it was justified by the lack of alternative options to obtaining support from government or other organizations. Emotional trauma from forced geographical displacement and abductions by the Lord’s Resistance Army had left profound impressions in many of the people in Gulu. This has added an extra barrier for people with disabilities when it comes to being able to live a full life where they can participate in their communities. As one participant from Gulu shared:

*‘Trauma and stress is very serious… you people need to address trauma so that our people begin to think positively and productively in order for them to participate fully in ensuring their personal health, the health of their families and for the community. Otherwise, all the good intentions will not yield good results’.*

## Discussion

There is an expectation that the post-2015 development agenda may redress long-standing political and resource deficits. There is also a need for progress-inclusive development practices that will be able to incorporate a wide array of voices, including those of older people as well as those from people with disabilities. Several reports support growing momentum for the post-2015 health and development goal agenda to ensure the prioritization of health inequities experienced by older persons, persons living with disabilities and others [14,29]. This can only be achieved through the effective engagement of communities in accountability and monitoring processes that are able to raise up priorities and concerns, as well as implement actions that will support the achievement of the post-2015 agenda [14].

Both sets of participants expressed that their needs are neglected by the Ugandan state, but also by society in general and by the health system more specifically. This led the participants to express feeling like outcasts as opposed to full members of their communities. The embodied stigma has led to a negative perception towards themselves and of their contribution to society [30]. For older persons, the feeling that they are considered superfluous may be reflective of a larger shift in African countries, where the growing number of younger people and the diminishing numbers of older ones impact traditional ways of life [31]. In regards to life at the village level, the feelings of rejection have isolated the members of these two groups from the benefits that living in a tight-knit community bring, namely a sense of belonging, solidarity and of being able to depend on each other to deal and solve life issues in a way that strengthens the bond between neighbors and families [32].

When it comes to access to health services, the societal changes the country has undergone has translated into lower levels of reported quality of life and a perception of poorer health outcomes for both groups of participants. The multiple reports of abuse towards both people with disabilities and older people in public health services include actions that go from the refusal of services up to sexual abuse. These experiences have a negative influence on health-seeking behavior and pushes population groups away from services that are badly needed [33]. In addition, they contribute to the perception that the health system is selective, exclusive and does not care for the population that is there to serve. Another major barrier to healthcare was communication. Participants from both groups faced this. People with disabilities may require additional support within the public health service to communicate with healthcare staff, as has been pointed out in previous studies [34]. Older participants reported that healthcare staff did not discuss with them what was happening or why they were in the health service. This is a common occurrence, not only among the participant groups in Kamwenge and Gulu, but also among people living with HIV, with indigenous populations and young people in many different regions of the world [5,35,36]. Studies from other parts of the world point to the need to develop more responsive and friendly services that can provide the communities they serve with the care they deserve [32,35].

As part of a comprehensive approach to dealing with the issues raised by both of these groups, it is necessary to implement not only essential health services but also mental health services that will be able to provide support [14]. This finding echoes the UN Special Rapporteur on the right to health’s report on stigma and mental health in which he notes, ‘*The World Health Organization recommends that mental health services, including support services, be based in the community and integrated as far as possible into general health services, including primary health care, in accordance with the vital principle of the least restrictive environment*’ [37]. This could come in the form of counseling for trauma, alcoholism and the effect of geographical displacement for not just people with disabilities and older people but also the general population. Participants also reported a lack of services to support people with disabilities to gain access to and maintain assistive devices.

### Study limitations

This study is one of the first to seek the perceptions of these two marginalized population groups in Uganda. It built strong relationships and rapport within the communities and sought to gain the perspectives of a wide range of respected members of the community and local community leaders. The focus group discussion in which they participated occurred as a one-off, large group discussion in each community. To gain a more in-depth understanding of the perspectives and experiences of the participants, smaller groups, which met more than once, may have been useful. However, this was beyond the project’s time and resources. Key informant interviews with community leaders were also conducted, building on the first phase and aiming to seek specific challenges for the people in their community.

## Conclusion

Our findings suggest that there is a strong likelihood that the right to health, as well as inter-related rights of persons with disabilities and older persons in Uganda are not being adequately protected, promoted and fulfilled in that country. Furthermore, our findings highlight the Ugandan government is not progressively realizing its right to health obligations in international law to either group, per the Government’s obligation to do so for both sets of participants pursuant to its ratification of the CRPD [38] and through its accession to the CESCR [9]. While the Ugandan government’s acts and/or omissions can be pointed to, the international community also has a legal obligation to support Uganda to implement the terms of both international treaties, particularly as Uganda is a low-income country [39]. Indeed, in a post-2015 world, there may be greater onus on both the international community and Ugandan government to support attainment of the essential health needs for both sets of communities given their vulnerable and marginalized position, and diminished health status in comparison to the general population.
